# Health Disparities of Sexual Minority Patients Following Prostate Cancer Treatment: Results From the *Restore-2* Study

**DOI:** 10.3389/fonc.2022.812117

**Published:** 2022-02-04

**Authors:** B. R. Simon Rosser, Elizabeth J. Polter, Kristine M. C. Talley, Christopher W. Wheldon, Ryan Haggart, Morgan Wright, William West, Darryl Mitteldorf, Michael W. Ross, Badrinath R. Konety, Nidhi Kohli

**Affiliations:** ^1^Division of Epidemiology and Community Health, School of Public Health, University of Minnesota, Minneapolis, MN, United States; ^2^Adult and Gerontological Health Cooperative, University of Minnesota School of Nursing, Minneapolis, MN, United States; ^3^Department of Social and Behavioral Sciences, College of Public Health, Temple University, Philadelphia, PA, United States; ^4^Department of Urology, University of Minnesota, Minneapolis, MN, United States; ^5^Department of Writing Studies, University of Minnesota, Minneapolis, MN, United States; ^6^Malecare Cancer Support, New York, NY, United States; ^7^Department of Family Medicine and Community Health, University of Minnesota Medical School, Minneapolis, MN, United States; ^8^Department of Urology, Rush Medical College, Chicago, IL, United States; ^9^Department of Educational Psychology, University of Minnesota, Minneapolis, MN, United States

**Keywords:** health status disparities, sexual minorities, healthcare disparity, prostatic neoplams, sexual dysfunction, physiological

## Abstract

**Purpose:**

The NIH has identified sexual and gender minority persons as a health disparity population but little is known about cancer outcomes in these populations. The purpose of this study was to identify disparities in sexual minority prostate cancer patient-reported outcomes, to examine within group differences, and to test for alternative explanations for identified differences.

**Materials and Methods:**

In 2019, we recruited 401 gay and bisexual prostate cancer patients into the *Restore-2* study, a randomized controlled trial of rehabilitation program tailored for sexual minority men.

**Results:**

Compared to the normative (heterosexual) EPIC sample, participants had significantly worse urinary, bowel and hormonal function, better sexual function, and no difference on bother scores. They also had worse depression and overall mental health, and worse physical, social/family, functional, prostate specific and overall well-being quality of life outcomes. Across measures, no differences by age, gay versus bisexual orientation, race/ethnicity, and relationship status were observed. Those who had hormonal treatment had worse sexual and hormonal function than those who had radiation or surgery only. Those with a longer time since treatment had better urinary function. Differences remained when participants were matched to normative samples on cancer stage and time since treatment.

**Conclusions:**

This, the largest study of sexual minority prostate cancer patients to date, confirms health disparities in prostate cancer quality of life outcomes. Findings appear reliable and robust. To improve the clinical care of prostate cancer, it will be important to address the health disparities experienced by sexual minority prostate cancer patients.

## Introduction

Sexual and gender minority (SGM) populations experience significant cancer health disparities (1), but are under-represented to entirely missing in research on cancer patients and survivors (1–5). According to a recent review by researchers at the National Cancer Institute (NCI), “SGMs face a disproportionate burden of cancer, yet little is known about the experience and needs of these underserved populations in cancer care delivery (3).”

Of the 3.1 million men diagnosed with prostate cancer in the US, about 63,000 are sexual minorities (6). Only six quantitative studies of sexual minority prostate cancer patients have been conducted (7–11); and of these, only three used standardized patient-reported outcome measures (11–13). Hart et al. (9) conducted a study of 92 North American gay and bisexual prostate cancer patients, diagnosed within the past four years (14). Participants were recruited online and through community advertising, confirmed eligible by telephone interview, and completed an online survey. Ussher et al. (15) conducted a study of 124 gay and bisexual prostate cancer patients living mainly in Australia, North America, or the United Kingdom (13). Participants were recruited using a mix of clinic and community outreach within Australia, and online internationally. The *Restore-1* study, conducted by our team in 2015-2016, comprised of 192 gay and bisexual and 1 transgender women prostate cancer patients living in North America (11). Participants were recruited from an online cancer site providing support services for sexual minority patients. Both the Hart et al. (14) and *Restore-1* (11) studies compared results to published norms (of predominantly heterosexual patients), while Ussher et al. (13) recruited a comparison sample of Australian heterosexual patients.

To measure treatment outcomes, Hart et al. (14) and *Restore-1* (11) used the Expanded Prostate Cancer Index Composite (EPIC-50). In both studies, sexual minority patients scored worse on urinary and hormonal function and better on sexual function than published norms (11, 14). Ussher et al. only used the sexual subscale, but also found better sexual function than published norms. For bother, the results were not consistent. Hart et al. reported worse urinary, bowel and hormonal bother, while *Restore-1* only found worse hormonal bother (11). For sexual bother, there was no consistency with studies reporting better (11), same (14), and worse (13) scores than the normative sample.

All the studies, using various measures, found sexual minority patients to have worse mental health than published norms. On physical health, results were inconsistent. Ussher et al., found no differences, while *Restore-1* reported better physical health than published norms. Ussher et al. was the only study to use the FACT-P, finding sexual minority patients scored worse on the emotional and day-to-day subscales (15).

There are three main limitations to these studies. First, all the studies had relatively small sample sizes which increases the risk of Type-2 error. Second, on several scales, there has been only one study, preventing researchers from assessing the replicability of findings. Third, none of the studies explored for alternative explanations for the observed differences.

The purpose of this study was three-fold. First, we sought to document the health disparities between sexual minority patients and published norms (for heterosexual patients). Second, we examined within group differences to identify sexual minority patients at higher risk of health disparities. Third, we tried to disprove the disparities by testing for two alternate explanations: namely that differences in stage of cancer and time since treatment might explain the results.

## Materials and Methods

### Participants

This paper describes the baseline survey results for all participants the *Resture-2* study, a clinical trial designed to test the effectiveness of an online rehabilitation program tailored for sexual minority prostate cancer patients. Participants needed to identify as gay, bisexual, or a man who has sex with men (regardless of whether they were currently sexually active) and live in the US. Transgender women were also welcome to participate, although none did. Enrollees had to be diagnosed with prostate cancer and either completed treatment at any point in the past, currently in treatment, or scheduled to receive treatment within two months of commencing the study. Appropriate to a rehabilitation study, participants also needed to report a current sexual and/or urinary problem. Implicit eligibility required participants to read English and be able to access intervention materials online. All data in this paper were taken from the baseline survey. The study was conducted under the oversight of the University of Minnesota Institutional Review Board.

A full description of the recruitment protocol has been published (16). To advance methods on this “hidden”, “difficult to recruit” population, we conducted a naturalistic, 3-arm, stratified prospective study to compare three recruitment strategies: (a) clinic based recruitment of prostate cancer patients from gay health and urology clinics; (b) directly from the gay community; and, (c) online recruitment (through cancer support, sex/dating, and social sites). For each strategy, we estimated time, workload, and direct costs involved. To study how recruitment strategy may affect sampling, we tested for retention rates, demographic and outcome differences across sites. From October, 2018 to August, 2019, participants were recruited mainly from the three online websites: a sex/dating site (*Scruff: n*=158), an online cancer support group network (*Malecare: n=89*) and a social site (*Facebook: n=66*). Participants were also recruited through gay media (*n*=39), word of mouth or other online sources (*n*=35) and least successfully from clinics (*n*=9). Five men from our prior study, *Restore-1*, also participated.

Prior to participation, each enrollee completed a vetting telephone interview to validate eligibility lasting about 20 minutes. Next, they completed an online consent process adapted from our prior research (17). We received a waiver of written consent for this online study.

After completing the consent process and baseline survey (from which all the data in this paper were taken), participants were randomized to either the online intervention or usual care. Participants were randomized 1:1 to either the intervention (an Internet-based, comprehensive sexual and urinary rehabilitation program) or the usual care control group. In order to ensure that both the control and intervention arms included enough recently treated men, randomization was stratified by time since prostate cancer treatment completed. Permuted block randomization in blocks of two ensured balance between the two arms of the study.

Our bio-behavioral intervention had seven key elements: *(1) PDE5-I drugs:* Participants with ED challenges were recommended to take 50mg sildenafil (i.e., Viagra^©^) orally, 3x per week for 2 years. These were provided at no charge by the study with a prescription from their physician. *(2) Pelvic floor exercises (a.k.a. Kegels):* To strengthen the *levator ani* muscle, to treat both urinary incontinence and climacturia, participants were instructed to do 10 quick contractions (2 sec. hold; 4 sec. relax) then one set of 10 long contractions (10 sec. hold; 10 sec. relax), repeated 3 times per day. To teach Kegels, we produced a video of a gay peer modeling how to do them. *(3). Vacuum pump and penile constriction rings:* All participants received a vacuum pump and “cock rings” to aid getting and maintaining erections. *(4) Anal dilators:* All participants received a set of 3 different sized butt plugs repurposed as anal dilators to treat pain in receptive anal sex. *(5) A Gay Man’s Guide to Good Sex after Prostate Cancer:* Online, participants had access to a comprehensive guide to restore functioning, including protocols to treat urinary incontinence, erectile dysfunction, anodyspareunia (painful receptive anal sex), and problems with arousal incontinence and climaturia. In addition, we produced videos modeling how sexual minority men with PCa deal with sexual challenges (e.g., disclosing PCa to a sex partner); a male couple discussing how they have good sex, post-treatment; a FAQ section where participants could ask questions and read answers from experts; and a tracking program where users could monitor their rehabilitation. *(6). Social support:* Given the lack of social support gay and bisexual men experience and our needs assessment results showing this as a priority, participants could access to a monitored noticeboard group where they posted questions to other peers and could respond. *(7). Coach:* Given stigma and lack of social support, participants could discuss their progress with a sexual health coach (study staff trained in motivational interviewing) every three months during the 24-month trial.

Of 461 participants who completed the screening and vetting process, 17 were excluded because they failed to meet the inclusion criteria, 42 declined consent or did not complete the baseline survey, and one duplicate response was excluded. Participants were compensated $50 for the baseline survey. The final sample comprised 401 participants.

### Measures

The survey was in English and comprised 338 questions. Skip and branch patterns were used to administer only those questions relevant to each participant.

*Demographics, Sexual Characteristics, Medical Information and Internet Use*. Demographic questions were adapted from the US Census and from the 2018 American Community Survey (18). Sexual and medical characteristics were based on the *Restore-1* study (11).

Prostate cancer treatment was investigated by asking participants to check which treatments they had undergone in nine categories, which at analysis were collapsed into: surgery only, radiation only, hormone therapy (in combination with surgery and/or radiation) and other. Participants reported their PSA levels at diagnosis, Gleason score (e.g., *3* + *3 (6)* or *3* + *4 (7)*) at diagnosis, and selected their stage (I, II, III, IV, or don’t know/don’t remember) using multiple choice menus. Gleason scores were then grouped according to grade groups (19).

*Disease Specific Quality of Life*. The Expanded Prostate Cancer Index Composite (EPIC-50) measures urinary, bowel, sexual, and hormonal symptom frequency and perceived bother. The EPIC-50 has acceptable scale and subscale reliability (*r*≥0.80) and internal consistency (*α≥* 0.82) (14, 20). All scales total 100, with higher scores indicating better functioning or less bother.

*Brief Symptom Inventory-1*. The BSI-18 assesses mental health in four domains: somatization, anxiety, panic, and depression. Each domain consists of six Likert-type items (scores: 0-24), which are summed to create a total score (score: 0-72). Higher scores indicate worse mental health. The scale has high internal consistency, with Cronbach’s Alpha coefficients ranging from 0.74 to 0.89 (21).

*Functional Assessment of Cancer Treatment-Prostate*. The FACT-P measures quality of life related to cancer and its treatment in four domains: physical, social/family, functional, and emotional well-being, plus a prostate cancer symptom score. Higher scores represent better quality of life. Cronbach’s alpha coefficients ranged from 0.65 (for the prostate cancer domain) to 0.89 for (FACT-P total) (22).

### Statistical Analysis

Patient reported outcomes were compared to the published EPIC-50, BSI-18, and FACT-P normative samples using *t*-tests (20–22). ANOVAs and *t*-tests were used to identify differences for quality-of-life measures across demographic and medical characteristics. When ANOVA findings were statistically significant, pairwise comparisons were conducted with Tukey adjustment for multiple comparisons. All reported *p* values were 2-sided. To correct for multiple comparisons, corrected *q*-values were calculated, and considered statistically significant if they had a false discovery rate (q-value) less than 0.05.

To test whether differences were due to cancer stage or time since treatment, we randomly selected a subset of participants to match the distribution of cancer stage among EPIC normative participants, and repeated this with time since treatment to match on recovery period. The data were analyzed using Stata version 16 (StataCorp, College Station, TX, USA). Due to a programming error, one item was omitted from the EPIC hormonal bother subscale in the survey. However, scores are considered valid if only one item out of six is missing (20).

## Results

The demographic, sexual, and medical characteristics of the participants are detailed in **Table 1**. To summarize, this sample of sexual minority patients living in the US was predominantly white, non-Hispanic (86.8%), gay-identified (92.5%) with a mean age of 63.5 years (SD=6.6). Mean years since diagnosis was 5.3 years (SD=4.8), with almost half (45.4%) within two years of diagnosis. Most participants had grade group 1 (N=70), 2 (N=106), or 3 (N=64) prostate cancer and were diagnosed Stage I or II. Most (58.1%) had undergone a radical prostatectomy, 19.0% radiation, 16.5% treatment involving hormone therapy, and 6.4%, other treatment.

**Table 1:** Demographic Characteristics of the Sample.

N	N	%
Age (N, %)		
40-49	8	2.0
50-59	112	27.9
60-69	222	55.4
70-79	57	14.2
80+	2	0.5
Ethnicity		
Hispanic/Latino	20	5.0
Not Hispanic/Latino	377	95.0
Race		
White	364	90.8
Black/African American	22	5.5
Asian	2	0.5
American Indian or Alaska Native	0	0
More than one	7	1.8
Other	6	1.5
Identity (N, %)		
Gay/homosexual	371	92.5
Bisexual	30	7.5
Current Relationship Status		
Single	114	28.5
Widowed, divorced or no longer in a relationship	37	9.3
Dating (men or women)	50	12.5
Married or in a long-term relationship (with a man)	185	46.3
Married or in a long-term relationship (with a woman)	14	3.5
Gleason Grade Groups		
1	70	17.5
2	106	26.4
3	64	16.0
4	17	4.2
5	33	8.2
Don’t know/Don’t remember	111	27.7
Stage at Diagnosis		
I	141	35.2
II	73	18.2
III	30	7.5
IV	18	4.5
Don’t know/Don’t remember	139	34.7
Treatment Category		
Radical Prostatectomy or cryotherapy (only)	233	58.1
External Beam Radiation (only)	76	19.0
Hormone therapy (Lupron) (with any combination of treatments)	66	16.5
Other^a^	26	6.5
Years Since the Initiation of Treatment		
<2 years	183	45.6
≥2 years	218	54.4

^a^“Other” includes prostatectomy plus radiation, focal laser ablation, dutasteride.

(N=401 gay and bisexual prostate cancer patients living in the US).

Results on the EPIC-50 were compared with the original normative sample (20) and *Restore-1* study (11) (see **Table 2**). As compared with the normative sample, *Restore-2* participants had worse urinary, bowel and hormonal function, better sexual function, and no differences on any of the bother scores (*p*<.05). *Restore-2* participants had significantly worse sexual function and bother, worse bowel function, but less hormonal bother than *Restore-1* participants (*p*<.05).

**Table 2:** Expanded Prostate Cancer Index Composite (EPIC) scores compared to a prior study in gay and bisexual prostate cancer patients as well as heterosexual normative validation samples.

N	*Restore-2*	*Restore-1*			Validation Sample (19)		
EPIC Domain^a^	401	193			252		
	Mean (SD)	Mean(SD)	p-value	q-value	Mean(SD)	p-value	q-value
Urinary	79.4 (17.3)	81.4 (19.4)	0.21	0.22	86.5 (15.9)	<0.001	0.001
Function							
Bother	74.7 (18.2)	74.5 (20.8)	0.90	0.66	75.8 (20.6)	0.48	0.46
Sexual							
Function	35.5 (21.2)	40.5 (23.6)	0.01	0.01	29.5 (23.8)	<0.001	0.002
Bother	39.2 (26.2)	55.0 (25.0)	<0.01	<0.01	41.1 (30.2)	0.40	0.45
Bowel							
Function	76.8 (9.4)	89.0 (12.5)	<0.01	<0.01	87.9 (14.3)	<0.01	<0.01
Bother	85.6 (15.4)	84.5 (16.7)	0.43	0.45	85.3 (19.0)	0.83	0.63
Hormonal							
Function	78.7 (16.2)	79.3 (18.1)	0.68	0.58	84.0 (15.9)	<0.01	0.001
Bother	88.3 (13.1)^b^	82.1 (18.1)	<0.01	0.001	88.7 (14.3)	0.71	0.58

^a^Each EPIC subdomain score ranges from 0 to 100, with higher scores indicating better quality of life (better function, or less bother).

^b^One item on the EPIC Hormonal Bother subscale was accidentally omitted from the survey.

**Table 3** compares the results of *Restore-2* with the with the original BSI-18 (21) and FACT-P (22) normative samples and with the Ussher et al. study (13). Compared to the normative sample, *Restore-2* participants had significantly higher (i.e. worse) scores on depression and overall mental health. Compared with Ussher et al., *Restore-2* scored significantly less (i.e., healthier) on somatization, depression and overall mental health. On the FACT-P, *Restore-2* participants scored significantly worse on all quality of life outcomes (except for emotional well-being) than the normative sample. *Restore-2* participants did not differ from the Ussher et al. sample on any FACT-P scores, except prostate cancer specific wellbeing where *Restore-2* participants had less symptoms affecting their quality of life.

**Table 3:** Brief Symptom Index-18 (BSI-18) and Functional Assessment of Cancer Therapy-Prostate (FACT-P) scores compared to previous studies in gay and bisexual prostate cancer patients as well as heterosexual validation samples.

N	*Restore-2*	Ussher et al.			Validation/Normative Sample		
	401	119			402 (BSI-18) (20) or 96 (FACT-P) (21)		
	Mean (SD)	Mean (SD)	p-value	q-value	Mean (SD)	p-value	q-value
BSI-18^a^						402	
Somatization	2.09 (2.42)	2.81 (4.12)	0.02	0.02	2.34 (2.99)	0.19	0.26
Anxiety	2.57 (3.47)	1.95 (2.58)	0.17	0.08	1.42 (2.72)	<0.001	0.09
Depression	3.62 (4.51)	4.65 (5.40)	0.03	0.03	1.55 (2.72)	<0.001	0.001
Panic	0.85 (1.67)	1.27 (2.34)	0.02	0.02	–^c^	–^c^	–^c^
Overall	8.28 (8.80)	10.7 (12.4)	0.02	0.02	5.54 (7.90)	<0.001	0.001
FACT-P^b^							
Physical well-being	23.4 (4.2)	23.9 (4.5)	0.26	0.11	26.2 (2.8)	<0.001	0.001
Social/Family well-being	18.9 (5.7)	18.2 (6.0)	0.25	0.11	23.5 (4.3)	<0.001	0.001
Emotional Well-being	17.5 (3.4)	17.1 (4.5)	0.30	0.13	15.5 (4.2)	<0.001	0.001
Functional Well-being	20.1 (5.5)	20.0 (6.0)	0.86	0.33	21.6 (5.2)	0.02	0.02
Prostate Cancer Specific Well-being	33.1 (6.8)	34.5 (7.3)	0.05	0.04	36.9 (6.6)	<0.001	0.001
Overall Well-being	112.9 (19.3)	114.0 (22.7)	0.60	0.11	130.5 (16.3)	<0.001	0.001

^a^BSI-18 subdomain scores range from 0-24, with higher scores indicating greater psychological distress. The BSI-18 score is a sum of the three subdomain scores, ranging from 0 to 72.

^b^FACT-P scores vary in absolute ranges, and higher scores indicate better quality of life.

^c^Panic subscale not included in norm manuscript.

### Differences Within Sexual Minority Prostate Cancer Patients

Among *Restore-2* participants, there were few demographic or treatment differences in patient-reported outcomes (see **Table 4**). The hormonal treatment group had worse scores on the EPIC sexual and hormonal subscales than those who had surgery only or radiation only. Those more than two years since treatment had better urinary scores than those who had had treatment more recently. There were no differences observed by age, race/ethnicity, relationship status, or gay versus bisexual sexual orientation.

**Table 4:** Bivariate analyses of quality of life scores by age, race/ethnicity, relationship status, sexual orientation, type of treatment, and time since treatment.

	EPIC^a^ Domain Scores				FACT-P^b^ Total	BSI^c^ Total
	Urinary Overall	Sexual Overall	Bowel Overall	Hormone Overall		
	Mean (SD)	Mean (SD)	Mean (SD)	Mean (SD)	Mean (SD)	Mean (SD)
**Age**						
<65	76.7 (16.4)	38.7 (22.0)	80.7 (11.8)	82.5 (13.9)	112.6 (19.9)	9.0 (9.4)
≥65	77.5 (15.4)	35.3 (22.1)	82.0 (10.7)	84.9 (13.6)	113.3 (18.6)	7.3 (8.1)
*p*-value	0.66	0.13	0.25	0.09	0.72	0.06
*q-*value	1.00	0.51	0.73	0.49	1.00	0.38
**Race/Ethnicity**						
White and Non-Hispanic	77.1 (15.7)	37.2 (21.7)	81.3 (11.0)	83.4 (14.0)	112.9 (18.9)	8.1 (8.6)
Non-White or Hispanic	76.9 (17.8)	38.5 (25.0)	81.0 (13.7)	84.4 (12.3)	113.0 (22.4)	9.4 (9.8)
*p*-value	0.95	0.72	0.88	0.57	0.97	0.39
*q-*value	1.00	1.00	1.00	1.00	1.00	0.97
**Relationship Status**						
Single, widowed, divorced, or no longer in a relationship	76.6 (16.3)	37.4 (22.7)	81.5 (11.7)	82.1 (14.1)	110 (19.8)	9.1 (9.0)
Dating	81.3 (15.8)	41.0 (21.3)	79.7 (12.8)	82.2 (15.1)	112 (22.4)	10.1 (10.7)
Married or in a long-term relationship with a man (n=185) or a woman (n=14)	76.2 (15.6)	36.5 (21.8)	81.4 (10.8)	84.9 (13.2)	115 (17.8)	7.2 (8.0)
*p*-value	0.12	0.44	0.06	0.14	0.01	0.04
*q-*value	0.51	1.00	0.38	0.51	0.13	0.36
**Sexual Orientation**						
Gay/Homosexual	77.3 (15.9)	37.5 (22.2)	81.4 (11.2)	83.5 (13.8)	113.2 (18.9)	8.3 (0.5)
Bisexual	74.0 (17.1)	34.9 (21.4)	79.5 (13.2)	83.2 (14.8)	110.1 (23.9)	8.3 (1.5)
*p*-value	0.32	0.54	0.38	0.92	0.41	0.99
*q-*value	0.83	1.00	0.97	1.00	0.97	1.00
**Type of Treatment**						
Radical Prostatectomy/Cryotherapy only	77.1 (1.0)	38.2 (21.7)*	82.4 (10.4)	85.5 (12.4)*	114.9 (19.2)	8.3 (9.5)
Radiation only	79.7 (1.8)	45.0 (22.5)*	79.0 (13.8)	84.8 (12.7)*	113.4 (19.3)	7.7 (6.8)
Hormonal treatment (in any combination)	75.1 (2.0)	25.9 (18.8)^†^	80.0 (10.0)	75.0 (16.8)^†^	107.2 (18.8)	8.3 (8.5)
Other*	74.0 (3.2)	35.3 (21.3)*^†^	80.1 (13.8)	83.6 (2.9)*	108.5 (19.0)	10.4 (10.0)
*p*-value	0.26	<0.01	0.11	<0.01	0.48	0.63
*q-*value	0.73	<0.01	0.51	<0.01	1.00	1.00
Time Since treatment start						
<2 years	75.3 (17.2)	38.8 (23.7)	80.5 (11.6)	83.0 (14.3)	113 (19.2)	8.0 (8.3)
≥2 years	78.7 (14.5)	35.9 (20.5)	81.9 (11.1)	83.9 (13.4)	113 (19.5)	8.5 (9.2)
*p*-value	0.03	0.20	0.21	0.49	0.82	0.58
*q-*value	0.33	0.73	0.73	1.00	1.00	1.00

(N=401 gay and bisexual prostate cancer patients living in the US).

^a^Each EPIC domain score ranges from 0 to 100, with higher scores indicating better quality of life (better function, or less bother). Each domain score is the average of its function and bother subdomain scores.

^b^The BSI-18 score is a sum of the three subdomain scores, ranging from 0 to 72, with higher scores indicating greater psychological distress.

^c^FACT-P scores vary in absolute ranges, and higher scores indicate better quality of life.

Cells containing the same symbol (*, ^†^) do not have statistically significant differences.

Having identified disparities in patient reported outcomes, we then tried to rule out alternative explanations (see **Supplementary Tables 1**, **2**). The differences in functioning remained significant, even after controlling for cancer stage and time since treatment.

## Discussion

The main finding from this study is that sexual minority prostate cancer patients experience significant health disparities. Compared with heterosexual patients, sexual minority patients score worse on EPIC-50 urinary, bowel, and hormonal functioning. While they score better on sexual functioning than heterosexual patients, both groups score poorly on this scale. Similarly, on the FACT-P, sexual minority patients have worse physical, social, emotional, prostate-specific and overall wellbeing. Sexual minority patients have worse overall mental health and possibly worse depression. Overall, these disparities appear robust, reliable and cannot be explained by the sexual minority participants having more advanced cancer or differences in time since treatment. And because the differences in EPIC were in function not bother, they cannot be explained away by stereotypes of sexual minority patients being more sensitive or emotional than heterosexual patients.

It is not obvious why the disparities between sexual minority and majority patients occur. That sexual minority men have better sexual function is likely due to differences in sexual behavior (e.g., more frequent masturbation), possibly motivation, and strategies men in same-sex relationships use to accommodate the sexual effects of treatment (e.g., non-monogamy, changes in sex roles) (11). Alternatively, a greater percentage of heterosexual patients than sexual minority patients may not be sexually active.

The worse scores on mental health are consistent with prior research (23), minority stress theory (24), and with sexual minority patients having less social support (25), and poorer experiences in treatment (3). In a recent survey of 112 urologists in the US, most providers said they do not ask about sexual orientation, are more comfortable discussing sex with heterosexual patients, lack knowledge about sexual minority patients, and feel inadequately trained in sexual minority health care (26). Heteronormative healthcare may contribute to the worse urinary, bowel and functioning scores, although the mechanism for this is not obvious.

The lack of within group differences suggests sexual minority prostate cancer patients are a more homogeneous group than heterosexual patients. We found no evidence of differences common in heterosexual patients, including no differences by age, and no marriage benefit in sexual outcomes.

Health disparities have important implications for clinical practice. Clinicians should note the sexual orientation datum in the patient’s electronic medical record or ask a patient his orientation as standard practice. When discussing treatment options, clinicians need to review the differential effects of treatment on insertive and receptive sexual functioning (6). In addition to sex, providers need to ask about urinary, bowel and hormonal function in sexual minority patients, and identify rehabilitation goals as appropriate. This may require additional time with sexual minority patients (6).

Clinicians also need to be cognizant of the additional mental health challenges this population experiences. Where providers are less comfortable in treating sexual minority patients, supplemental training should be provided. Some providers may feel they strive to provide the same high quality care to all patients (6). Such providers need to be educated in the difference between equality and equity in healthcare (27). If a minority consistently experiences worse outcomes, it suggests something (or multiple things) in the healthcare system is failing these patients.

There are three main limitations to consider in this study. First, this study (and also Hart et al. and *Restore-1*) relied on published norms for the comparison. Some scales were developed using small samples and they may be dated. While Ussher et al. overcame this by recruiting a comparative heterosexual sample, their sexual minority and heterosexuals were recruited differently introducing a confound. Second, all the sexual minority studies used cross-sectional surveys, preventing imputation of causality. For example, we cannot know whether mental health disparities in participants preceded their diagnosis, or whether treatment caused, exacerbated or decreased any preexisting vulnerability. Third, the sexual minority samples in all the studies to date are very homogeneous, comprising mainly white, gay-identified, HIV-negative, cisgender men. Caution should be exercised generalizing beyond these demographics to other sexual minority patients, and to gender minority patients as well.

To advance research on disparities, we need four types of studies. Prospective controlled studies in both heterosexual and sexual minority patients would enable us to infer causation while confirming disparities and updating norms on the key prostate cancer scales. A qualitative investigation is needed to identify what sexual minority and heterosexual patients do post-treatment to explain the improved sexual functioning in sexual minority men. And, we need studies of clinicians and clinical systems, including evaluation of training programs, to improve provision of sexual minority healthcare. Finally, we also need studies of best practices to transform clinical care to be more culturally responsive to the needs of sexual minority patients.

## Conclusion

In the largest study of sexual minority prostate cancer patients to date, we confirm multiple health disparities in outcomes for sexual minority prostate cancer patients. As compared to published norms for heterosexual patients, sexual minority prostate cancer patients suffer worse urinary, bowel, and hormonal functioning, worse prostate quality of life and worse mental health, but better sexual functioning. We also observed few within group differences across sexual minority patients. To improve clinical care, it will be important to address the health disparities of sexual minority prostate cancer patients.

## Data Availability Statement

The raw data supporting the conclusions of this article will be made available by the authors, without undue reservation. At the end of the study, data will be available at the Data Repository of the University of Minnesota (DRUM).

## Ethics Statement

The studies involving human participants were reviewed and approved by Institutional Review Board, University of Minnesota. The patients/participants provided their written informed consent to participate in this study.

## Author Contributions

Conceptualization: BR, KT, CW, DM, MR, BK, and NK. Methodology: BR, NK, EP, and MW. Software N/A. Validation: NK and EP. Formal Analysis: NK, EP, and MW. Investigation: BR, EP, MW, RH, and NK. Resources: Not applicable. Data Curation: RH, MW, and NK. Writing – Original Draft: BR, EP, and KT. Writing – Review & Editing: All authors. Visualization – Not applicable. Supervision: BR and NK. Project administration: BR, BK, and NK. Funding acquisition: BR, KT, CW, WW, DM, MR, BK, and NK. All authors contributed to the article and approved the submitted version.

## Funding

This study is supported by funding from the National Cancer Institute (NCI) [1R01CA218657; PI: Rosser]. The content is solely the responsibility of the authors and does not necessarily represent the official views of the National Institutes of Health.

## Conflict of Interest

The authors declare that the research was conducted in the absence of any commercial or financial relationships that could be construed as a potential conflict of interest.

## Publisher’s Note

All claims expressed in this article are solely those of the authors and do not necessarily represent those of their affiliated organizations, or those of the publisher, the editors and the reviewers. Any product that may be evaluated in this article, or claim that may be made by its manufacturer, is not guaranteed or endorsed by the publisher.

## Abbreviations

BSI18, Brief Symptom Inventory-18; EPIC, Expanded Prostate Cancer Index Composite; FACT-P, Functional Assessment of Prostate Cancer Treatment – Prostate; SGM, Sexual and Gender Minority.
