# Supplementary material for: Health Disparities of Sexual Minority Patients Following Prostate Cancer Treatment: Results From the Restore-2 Study
Source: Front Oncol. 2022 Feb 4;12:812117. doi: 10.3389/fonc.2022.812117 (PMC8854183; doi:10.3389/fonc.2022.812117)
Supplement: Supplementary file 1 [file Table_1.docx]

**Supplementary Table 1: Comparison of EPIC-50 scores between the Normative Heterosexual Sample and a Random Subsample of *Restore-2* Participants Matched on Stage of Cancer at Time of Diagnosis**

|  | **Wei et al.** | | ***Restore-2*^a^** | | ***p*-value** | ***q*-value** |
| --- | --- | --- | --- | --- | --- | --- |
| N | 252 | | 158 | |  |  |
|  | Mean | SD | Mean | SD |  |  |
| Epic |  |  |  |  |  |  |
| Urinary Function | 86.5 | 15.9 | 78.8 | 17.2 | <0.001 | **0.003** |
| Urinary bother | 75.8 | 20.6 | 73.6 | 18.7 | 0.28 | 0.23 |
| Sexual Function | 29.5 | 23.8 | 36.3 | 19.7 | 0.003 | **0.004** |
| Sexual Bother | 41.1 | 30.2 | 37.6 | 25.2 | 0.23 | 0.23 |
| Bowel Function | 87.9 | 14.3 | 76.9 | 8.9 | <0.001 | **0.003** |
| Bowel Bother | 85.3 | 19 | 84.9 | 15.1 | 0.82 | 0.58 |
| Hormonal Function | 84 | 15.9 | 78.2 | 16.7 | 0.0005 | **0.003** |
| Hormonal Bother | 88.7 | 14.3 | 88.3 | 13.0 | 0.78 | 0.58 |
| ^a^ Random subsample of *Restore-2* participants matched on Treatment stage with Wei et al.’s normative sample. | | | | | | |
