# Supplementary material for: Health Disparities of Sexual Minority Patients Following Prostate Cancer Treatment: Results From the Restore-2 Study
Source: Front Oncol. 2022 Feb 4;12:812117. doi: 10.3389/fonc.2022.812117 (PMC8854183; doi:10.3389/fonc.2022.812117)
Supplement: Supplementary file 2 [file Table_2.docx]

**Supplementary Table 2: Comparison of EPIC-50 scores between the Normative Heterosexual Sample and a Subsample of Restore-2 Participants Matched by Mean and Standard Deviation months since treatment**

|  | **Wei et al.** | | **Restore-2^a^** | | ***p*-value** | ***q*-value** |
| --- | --- | --- | --- | --- | --- | --- |
| N | 252 | | 205 | |  |  |
| Months since diagnosis (SD) | 25.7 (13.0) | | 25.6 (12.8) | |  |  |
|  |  | |  | |  |  |
|  | Mean | SD | Mean | SD |  |  |
| Epic |  |  |  |  |  |  |
| Urinary Function | 86.5 | 15.9 | 79.1 | 16.8 | <0.001 | **0.001** |
| Urinary bother | 75.8 | 20.6 | 73.9 | 18.3 | 0.30 | 0.32 |
| Sexual Function | 29.5 | 23.8 | 36.3 | 21.1 | 0.002 | **0.004** |
| Sexual Bother | 41.1 | 30.2 | 39.7 | 27.0 | 0.61 | 0.47 |
| Bowel Function | 87.9 | 14.3 | 76.6 | 10.1 | <0.001 | **0.001** |
| Bowel Bother | 85.3 | 19 | 86.2 | 14.6 | 0.58 | 0.47 |
| Hormonal Function | 84 | 15.9 | 78.3 | 16.1 | 0.002 | **0.004** |
| Hormonal Bother | 88.7 | 14.3 | 88.1 | 12.4 | 0.64 | 0.47 |
| ^a^ Random subsample of Restore-2 participants matched on Treatment stage with Wei et al.’s normative sample. | | | | | |  |
